# Young children's emotional responses to new peers: The roles of behavioural inhibition and relationships

**DOI:** 10.1093/chidev/aacag030

**Published:** 2026-03-20

**Authors:** Linda Sosa-Hernandez, Sebastian P Dys, Kathryn A Degnan, Nathan A Fox, Heather A Henderson

**Affiliations:** Department of Psychology, University of Guelph, Guelph, Ontario, Canada; School of Psychology, University of Canterbury, Upper Riccarton, Christchurch, New Zealand; Department of Psychology, The Catholic University of America, Washington, D.C., United States; Department of Psychology, University of Maryland, College Park, MD, United States; Department of Psychology, University of Waterloo, Waterloo, Ontario, Canada

**Keywords:** affect expressions, peer interactions, relationships, reciprocity, behavioral inhibition

## Abstract

This study examined the extent to which young children's affect expressions during interactions with new peers are attributable to individual traits versus unique relationships, and how behavioral inhibition (BI) relates to these expressions. Using a longitudinal dyadic design collected between 2003 and 2008, 291 children (*M*_age_ = 24 months at baseline, 60% female, 69.7% White) were observed playing with unique age-and gender-matched peers annually from 24 to 60 months of age. Variability in children's affect expressions was primarily accounted for by the unique relationship instead of individual differences. Children higher in BI displayed less positive affect (*B* = −0.30) and elicited less negative affect (*B* = −0.15) from peers. Findings underscore how both individual characteristics and relational processes shape early peer interactions.

From a young age, peer interactions shape children's social and emotional development, helping them learn about morality, understand emotions, build social skills, and develop a sense of belonging (Baumeister & Leary, 1995; Hughes & Dunn, 2007). Peer relationships also protect children from being victimized and facing mental health challenges (Baardstu et al., 2020). Therefore, understanding what drives the formation of peer relationships in young children is essential. Research has typically focused on individual traits, such as a child's temperament. For example, behavioral inhibition in early childhood is linked to fewer and lower quality peer interactions (Rubin et al., 2006). More recent studies show that peer interactions are also shaped by the relationship itself, including the peer's characteristics and the unique bond between two children (e.g., Hubbard et al., 2023). Consider a child who tends to be inhibited and show negative emotions around other children. If they meet another child who is eager to make friends (e.g., smiling and inviting them to play) the inhibited child might act friendlier and respond with more positive emotions than usual. In this way, children's responses are shaped by both their temperament and the specific peer interaction. We believe that these work together to influence children's responses, and ultimately whether an acquaintance turns into a friend.

Research has started to elucidate how children's emotions and behaviors are influenced by their peers within structured settings, such as guided pretend play and speech tasks, during early (Gibson et al., 2020; Jaggy et al., 2020) and middle childhood (Dys et al., 2023; Moore et al., 2023). However, there remains much to be understood about how these unfold during unstructured interactions and over time, especially during early childhood (ages 2–5). This developmental period is marked by substantial growth in children's cognitive, motor, and communication skills that allow for a wider range of emotional expressions and behaviors during peer interactions. Unstructured interactions are also important at this stage, as children frequently engage in free play with peers not only during play dates but also as a core part of daycare and preschool programs. We used a longitudinal dyadic design called one-with-many to partition the relative contributions of individual and relationship processes on children's emotional expressions with new peers. We also examined how an aspect of children's temperament (i.e., behavioral inhibition) related to their own and their partner's affect expressions.

## Emotional expressions as social signals

Children differ considerably in their tendencies to express positive and negative emotions during social interactions, and these tendencies provide important cues to others about their openness to friendship (Camras, 2022). Functionalist perspectives of emotion posit that affect expressions serve both affiliative and distancing social functions (Barrett & Campos, 1987; Fischer & Manstead, 2008). Some children have a greater tendency to express positive affect, like smiling, which is generally thought to promote the formation, solidification, and maintenance of close social relationships (Fredrickson, 2001; Stifter et al., 2020). Across the lifespan, displaying positive affect is linked to better relationship quality (Griffith et al., 2019; Ramsey & Gentzler, 2015), increased peer liking (Dougherty, 2006), and greater motivation to connect with a previously unfamiliar social partner (Pearlstein et al., 2019). Conversely, other children are more prone to expressing negative affect, such as frowning, which can narrow the range of their behaviors and create a sense of distance between themselves and a potential new friend (Fischer & Manstead, 2008). A longitudinal social network study found that 3- to 4-year-old children who displayed more negative affect with classmates were less likely to engage in social play with the same peers eight months later (Neal et al., 2017). These social functions of positive and negative affect are consistent with the broaden-and-build theory (Fredrickson, 2001), which emphasizes that positive emotions help children try new behaviors and build lasting social resources that support relationship development over time, whereas negative emotions may restrict behavior and reduce opportunities for social connection.

Children's emotional expressions are also influenced by their interaction partner's characteristics and the unique qualities of their relationship with that person. To date, only a single study has differentiated the unique contributions of individual traits and the relationship processes in shaping children's emotional expressions during peer interactions. Hubbard et al. (2023) utilized a round-robin design to partition the contributions of individual traits and relational factors in explaining 9-year olds’ emotional expressions, such as happiness, sadness, anger, anxiety, and neutral affect, during structured tasks with new peers (including a frustration task and a cooperative planning task). In this study, variation in children's emotional expressions was attributed to individual differences (19%–44% of the variation), characteristics of their interaction partners (20%–39%), and unique aspects of the specific dyadic relationship (6%–62%). This highlights that both personal traits and the nature of the relationship influence children's emotional responses during structured tasks with new peers at the age of 9. Yet, it remains unknown how these influences manifest among younger children or in less controlled situations, like free play with a new peer.

## Emotional concordance

Children often show emotional concordance by matching each other's emotional displays, such as two peers smiling at the same time (Hess & Fischer, 2014). When children match each other's emotional expressions, it may reflect how in sync they are emotionally and how similarly they feel about the interaction (Hess & Fischer, 2014). Research shows that individuals align their emotional expressions throughout the life course, from infancy to adulthood (Lindsey & Berks, 2019; Moed et al., 2017; Thomassin & Suveg, 2014). In young children, there is evidence that infants and preschoolers can catch others’ emotions through facial expressions, vocal tone, physiology, and behavior in both peer and caregiver–child contexts (e.g., Geangu et al., 2010; Waters et al., 2014). Among school-age children, children's overall levels of expressed happiness, sadness, anger, or neutrality during structured tasks were associated with their peers’ expressions, particularly for happy and neutral expressions (Hubbard et al., 2023). In adults, research shows that when positive or negative affect is experimentally induced (e.g., by watching a video), those in a negative affective state display greater contagion to angry faces, whereas those in a positive state display greater contagion to happy faces (Pinilla et al., 2020). These findings suggest that emotional contagion may be valence-dependent, with positive and negative affect following distinct transmission patterns. Much of this prior work has used different terms and operationalizations, such as reciprocity, concordance, and contagion, to describe how emotions are aligned between partners, and it has typically examined these dynamics within a single dyad. We examine the overall association between children's affective expressions and those of their peers across multiple dyadic interactions with different partners in early childhood. Looking across multiple dyads allows us to test whether children's emotional expressions align more generally across interactions and to gain a broader understanding of the overall emotional concordance or tone of early peer encounters.

## Applying the one-with-many model to peer interactions

The current study uses a “one-with-many” (OWM) model to assess various one-on-one interactions that a focal child (i.e., the “one”) has with several different unfamiliar peers (i.e., the “many”; Kenny & Kashy, 2011). The OWM combines aspects of both the actor–partner interdependence model and the social relations model but differs in that a focal child interacts with *multiple* partners who do *not* interact among themselves. OWM allows us to disentangle the relative contributions of individual and relationship factors in children's emotional expressions by partitioning the variance of actor, partner, and relationship components (see Figure 1). The model also captures the overall degree of association between children's and their partners’ affective expressions, which may reflect the general emotional concordance or tone within the interaction. Beyond these components, the OWM design can also incorporate focal child, partner, or dyad-level characteristics. For example, we included age to test for potential developmental differences in children's emotional expressions across early childhood. Thus, applying the OWM approach to early peer interactions may help us understand the extent to which children's emotional expressions with new peers reflect stable individual dispositions versus being shaped by the specific peer relationship. If children's emotional expressions are partly relationship-specific, this may suggest that developmental science and practice could benefit from greater attention to dyad-level factors, such as how well two children fit together, when studying relationship formation and positive peer engagement. This approach also captures how child-, partner-, and dyad-level characteristics may shape the emotional climate of initial peer interactions.

**Figure 1:**
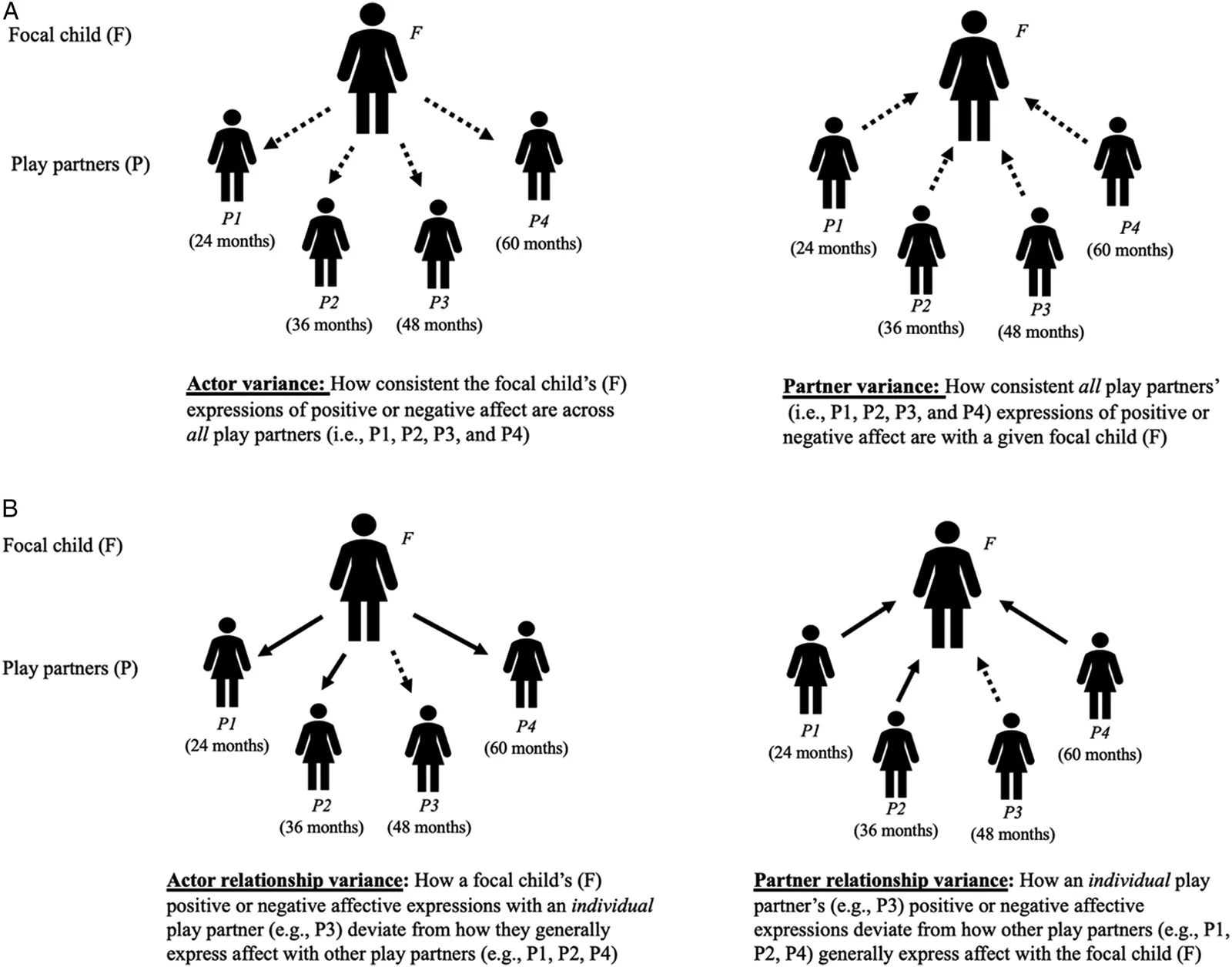
Description the actor, partner, and relationship variance components of the one-with-many model.

## Behavioral inhibition and emotional expressions: actor and partner effects

While many children eagerly approach new peers, those with behaviorally inhibited temperaments find such interactions anxiety-provoking. Behavioral inhibition (BI) is a temperament trait marked by intense discomfort toward new people and settings (Coplan and Armer, 2007; Fox et al., 2001; Kagan et al., 1987), which remains relatively stable across toddlerhood and childhood (Pérez-Edgar & Fox, 2005). Children with higher levels of BI tend to show more social hesitation, withdrawal, and reduced emotional expressivity with unfamiliar peers (Degnan et al., 2014; Kochanska & Radke-Yarrow, 1992). Not only do highly inhibited children struggle to engage with new peers themselves but they are also perceived more negatively by peers (Coplan & Armer, 2007; Zava et al., 2020) and experience more social rejection, exclusion, and victimization than their less inhibited counterparts (Rubin et al., 2009).

Central to relationship models of development is the notion that temperament, such as child's level of BI, influences both a child's own *and* their social partner's thoughts, behaviors, and emotions (Caspi et al., 1989; Henderson et al., 2018; Hinde, 1997). These evocative effects are at the core of relationship formation and may influence the social experiences of children high in BI (Burgess et al., 2006; Pedersen et al., 2007). While previous research has linked BI with higher negative and lower positive expressivity in children and adolescents, based on self, parent, and teacher reports, these trait-based assessments often mask the nuances of emotional expressions as they unfold during specific interactions (Eggum et al., 2012; Eisenberg, 2001; Viana et al., 2017). Observational studies have begun to examine how BI is related to children's own and their partner's emotion expressions in the moment, such as during initial encounters with a new peer (Almas et al., 2011; Sosa-Hernandez et al., 2023; Vallorani et al., 2022). For example, Vallorani et al. (2022) found that among children aged 5–7 years who were paired with unfamiliar, age- and sex-matched peers for a 5-min free play session, those higher in BI were less likely to express positive affect when their partner expressed positive affect, whereas BI was unrelated to their expressions of negative affect. Our study advances existing research by observing children with BI as they interact with four different unfamiliar peers. This approach allows us to explore whether the effects of BI on children's own emotions and those of their new peers remain stable across various partners and time.

## The present investigation

Guided by key emotion and relationship theories (Fischer & Manstead, 2008; Hinde, 1997) and research (e.g., Hubbard et al., 2023) highlighting the importance of both individual and relational processes in social interactions, we examined young children's emotional expression during initial peer interactions. A one-with-many design was used to follow each child annually from 24 to 60 months as they played with different, unfamiliar peers. We investigated how much of children's emotional expressions with new peers could be explained by their individual traits versus the specific relationship across early development (Aim 1). Based on Hubbard et al. (2023), we hypothesized that children's emotional expressions with peers would be accounted for by both individual differences *and* relational factors. However, because only one prior study has investigated the relative contributions of individual and relational factors in a peer relationship context, the question of which would account for more variance was considered exploratory. We also explored whether and to what degree children's emotional expressions were associated with those of their peers (Aim 2). Prior work suggests that the strength of these associations may vary by emotional valence, with peer dyads showing the strongest correlations for happy expressions in school-age children (Hubbard et al., 2023), whereas adults show stronger correlations for negative affect expressions (Pinilla et al., 2020). Given these mixed findings across developmental stages, we examined this question as an exploratory aim. We further examined whether children's BI was linked to their own and their partners’ emotional expressions (Aim 3). Based on prior evidence linking BI to children's own emotional expressions (e.g., Viana et al., 2017), we hypothesized that children higher in BI would display fewer positive and more negative affect expressions across unfamiliar partners. In contrast, because few studies have examined how BI relates to peers’ affective expressions during live interactions (e.g., Vallorani et al., 2022), we treated this aim as exploratory. This study was not preregistered.

## Method

### Participants

Participants were drawn from a larger longitudinal study examining children's temperament and social-emotional development. The present study included 291 focal children (156 females; 141 males) who were gender-and age-matched with a different unfamiliar play partner (who was not part of the initial sample) at 24 (*M* = 25.83, *SD* = 2.21), 36 (*M* = 37.24, *SD* = 1.56), 48 (*M* = 49.31, *SD* = 1.26), and 60 (*M* = 63.51, *SD* = 3.85) months of age. Of note, most unfamiliar play partners (72.2%) participated in more than one dyad; however, no focal child was paired with the same partner more than once. The dyadic visits relevant to the current study were conducted between 2003 and 2008. The ethnic composition of the mothers of focal children was: 69.7% White, 16.6% Black, 7.2% Hispanic, 3.1% Asian, and 3.4% other ethnicities. Mothers were predominately highly educated, with 77.6% earning college or graduate degrees, 16.2% earning high school diplomas, and 5.5% achieving other qualifications (0.7% did not report). Focal children and play partners did not differ on age, maternal education, maternal ethnicity, or their affect expressions during free play at any timepoint (*p*s ≥ .060). Additional sociocultural information (e.g., family income, family structure, language, religion, and generational status) was not collected.

Families with young infants were initially recruited using commercially available mailing lists in a large metropolitan area of the Mid-Atlantic region of the United States. Eligibility criteria included infants being full-term at birth with no serious medical or developmental problems. A total of 779 four-month-old infants were screened during an in-laboratory observation of their positive (i.e., higher motor activity and positive affect) and negative reactions (i.e., higher motor activity and negative affect) to novel auditory and visual stimuli. The final sample of focal children was enriched to capture the full range of temperamental reactivity, from negative to positive. Focal children were not selected based on their level of BI, which was measured at 24 and 36 months (see Measures below). In the current study, BI was analyzed as a continuous variable. Unfamiliar play partners were recruited from the community at each age without consideration of their temperamental reactivity. In all dyads, the “focal child” refers to the longitudinally followed participant, whereas the “play partner” refers to the unfamiliar peer. Only focal children's behavioral inhibition was assessed, and pairing at each visit was based solely on age and gender. Thus, both children could exhibit the full range of temperament, and dyads were therefore treated as statistically indistinguishable in analyses. For full details on the screening and recruitment protocol see Hane et al. (2008). All study procedures were approved by the Institutional Review Board (IRB) at the University of Maryland.

### Measures and procedures

#### Behavioral inhibition of focal children (24 and 36 months)

At 24 and 36 months of age, focal children's BI was assessed using maternal report and an in-laboratory observation of children's behavior in response to unfamiliar stimuli.

##### Maternal report of BI

Mothers completed the Toddler Behavior Assessment Questionnaire (TBAQ; Goldsmith, 1996), a 111-item parent-report measure that assesses children's temperament across six dimensions. Mothers rated how often their child exhibited the behaviors in each item during the past month on a 7-point Likert scales ranging from 1 “Never” to 7 “Always”. Given its relevance to BI, only the social fearfulness subscale was included in the present study. The social fearfulness subscale measures children's inhibition, distress, and shyness in novel and ambiguous situations (e.g., “If a stranger came to your home or your apartment, how often did your child: allow her/himself to be picked up without protest?”). The TBAQ has established convergent validity and reliability with children aged 16–36 months, and in the present study, the social fearfulness subscale had good internal consistency (α_24mo_ = .83; α_36mo_ = .88). Maternal reports of BI at 24 and 36 months were correlated (*r* = .577, *p* < .001).

##### Observations of BI

Focal children and their mothers also completed laboratory tasks designed to measure children's responses to unfamiliar social and non-social stimuli. The unfamiliar stimuli presented in these tasks included an adult stranger, a robot, and an inflatable tunnel. For each task, children's latency to vocalize, latency to approach/touch the stimuli, and proportion of time spent near their mother were coded reliably (ICC_mean_ = .92; ICC_range_ = .72–1.00). A BI composite was computed at each age by averaging the standardized scores of children's latency and proximity codes across tasks. Children's scores across the behavioral tasks were significantly correlated (*r*_mean_ = .40; ICC_range_ = .34−.48) and together engendered composites of acceptable reliability at each age (ICC_24mo_ = .60; ICC_36mo_ = .67). BI composites at 24 and 36 months of age were correlated (*r* = .33, *p* < .001).

##### BI composite

To derive a multi-method and multi-timepoint measure of BI, we aggregated maternal-reports and observations of BI across 24- and 36-month assessments. This overall BI composite is well-established in other studies using the present sample (e.g., White et al., 2011) and demonstrates acceptable reliability (ICC = .67).

### Affect expressions of focal children and play partners (24, 36, 48, and 60 months)

In laboratory visits separate from the individual BI assessments, focal children were paired with a different same-gender and same-age unfamiliar play partner, and dyads were observed during 10-minutes of unstructured play at 24, 36, 48, and 60 months of age. During the unstructured play, children were in a large room that contained a variety of age-appropriate toys. At the 24- and 36-month-old visits, mothers were present in the playroom and sat in chairs in two different corners of the room. Two trained observers coded the frequency and intensity of both children's positive and negative affect expressions on a 7-point scale ranging from 1 “Absence or few displays of affect” to 7 “Continued expression of intense affect”, based on verbal and behavioral attributes in 2-minute epochs. Positive affect was defined by displays of happiness (e.g., a smile, excited tones). Negative affect was characterized by displays of sadness and anger (e.g., a frown, negative tones). Inter-rater reliability was computed on 23% of the sample at each age for positive (ICC_mean_ = .80; ICC_range_ = .74−.85) and negative affect (ICC_mean_ = .81; ICC_range_ = .74−.89). Ratings were averaged across all five epochs.

### Data analytic plan

#### Data preparation procedures

Prior to conducting analyses, outliers were screened using criteria from West et al. (1995). For missing data, we tested whether children with available data differed from those without on demographic variables, and any variables showing differences were included as covariates. Further, multilevel modeling with a maximum likelihood estimator was used because it retains participants with missing data in longitudinal designs when the data are missing completely at random (MCAR) or missing at random (MAR; Snijders & Bosker, 1993). We followed Enders and Tofighi's (2007) recommendations for centering predictors. Accordingly, BI was grand-mean centered. Dyad age was calculated as the mean age of both children at each visit and centered at the first assessment (24 months). We elected to use dyad mean age in our analyses because children within each dyad were closely age-matched (within six months). Additionally, analyses entering focal children's and play partners’ ages separately did not meaningfully change the pattern or interpretation of the results.

#### One-with-many analyses

The one-with-many model was used as a theoretical and methodological framework to guide our analytic approach (Kenny et al., 2011). Multilevel modeling was used because it accounts for the dyadic structure of the present data (i.e., play partners nested within a focal child; Kenny et al., 2011). Failing to do so violates assumptions of independent observations and can bias statistical tests of significance. Members of the dyad in our study were indistinguishable as children were age- and gender-matched and thus, there was no meaningful variable to categorize them into distinct groups, as would be the case with parent–child dyads. Since we observed the positive and negative affect of both focal children and play partners, our study had a reciprocal design. Multi-level modeling for a reciprocal OWM is based on the two-intercept approach, in which two dummy variables are created to denote the person that provided the outcome score (Bolger & Laurenceau, 2013). This approach allows separate intercepts to be estimated for the focal child and play partners.

Figure 1 provides a summary of the components estimated by a OWM model. Our OWM models included fixed effects of dyad age, dyad gender, focal child ethnicity, play partner ethnicity, and focal child BI. We modeled positive and negative affect using two separate models because theory and prior research suggest they serve distinct functional roles in social behavior (Barrett & Campos, 1987) and show different patterns of concordance between partners (e.g., Hubbard et al., 2023; Pinilla et al., 2020). Also in our sample, correlations between positive and negative affect indicated they were largely independent dimensions (see Table 1), with no significant associations except at 48 months. To examine the relative contributions of individual and relational factors to children's affective expressions, we used the OWM model to partition variance in positive and negative affect into actor, partner, and relationship components. To explore the degree to which children's affective expressions were associated with those of their play partners (Aim 2), we used the OWM to estimate actor–partner reciprocity correlations for positive and negative affect. To test whether focal children's BI was linked to their own and their partners’ positive and negative affect expressions (Aim 3), we included BI as a fixed effect. Data were analyzed using R, Version 4.0 (R Core Team, 2018), with the *nlme* package.

**Table 1:** Descriptive statistics and pairwise correlations for behavioral inhibition at 24 and 36 months and children's observed affect at 24, 36, 48, and 60 months.

Variable	*n*	*M (SD)*	*ICC* ^a^	Intercorrelations^b^								
				1.	2.	3.	4.	5.	6.	7.	8.	9.
1. Behavioral Inhibition	268	−.005(0.47)	—	—								
Positive Affect												
2. 24 months	418	3.07(1.42)	.37***	−.24**	—							
3. 36 months	424	2.57(1.21)	.48***	−0.01	.12	—						
4. 48 months	404	3.47(1.21)	.52***	−.15*	.15*	.13	—					
5. 60 months	394	4.01(1.15)	.41***	−.16*	.02	.07	.21**	—				
Negative Affect												
6. 24 months	418	2.32(1.40)	.19**	−.03	−.07	.11	−.08	−.02	—			
7. 36 months	424	1.57(1.09)	.08	−.08	.06	.02	.23**	.14	.10	—		
8. 48 months	404	2.41(1.12)	.33***	−.03	−.04	.02	.14*	.08	.001	.003	—	
9. 60 months	394	2.39 (1.20)	.45***	.01	.05	.10	.10	.17*	.05	.20**	.03	—

aGriffin and Gonzalez's (1995) pairwise method was used to calculate (a) intraclass correlations (ICCs) between the focal child and the play partner on the same affect measures and (b) intercorrelations among the focal children's own scores across 24, 36, 48, and 60 months. In all cases significance levels were adjusted for the level of dependence observed among dyad members. *p < .05; **p < .01; ***p < .001.

## Results

### Descriptive statistics and preliminary analyses

Descriptive statistics including means, standard deviations, and correlations, for BI and positive and negative affect at each age are provided in Table 1. To account for the interdependence between dyad members on study measures, we used the pairwise method which adjusts the significance level of correlations by applying a *r*-to-*z* transformation (Griffin & Gonzalez, 1995). All study variables demonstrated acceptable normality and no outliers, except for negative affect at 36 months, which was positively skewed and thus winsorized to fall within three standard deviations (West et al., 1995). To examine overall age-related differences in children's affect expressions across the four timepoints, we conducted repeated measures ANOVAs. Results showed significant increases in both positive affect, *F*(3, 444) = 47.15, *p* < .001, ηp² = .24, and negative affect, *F*(2.84, 419.79) = 28.50, *p* < .001, ηp² = .16. For positive affect, scores at 36 months were significantly lower and scores at 60 months significantly higher than all other timepoints (*p*s < .01). For negative affect, scores at 36 months were significantly lower than all other timepoints (*p*s < .001), whereas scores at 24, 48, and 60 months did not differ.

### Missing data

Data were missing on the BI and observational affect measures (i.e., positive and negative affect). Of the original 291 focal children, 23 (i.e., 8%) had missing BI data. Missing data on the BI measures did not differ by maternal education, child age, child gender, positive affect, or negative affect at any timepoint (*p*s > .056). However, focal children with available BI data were more likely to have White mothers versus mothers from another ethnic group, χ^2^ (1) = 12.64, *p* < .001. Consequently, ethnicity was included as a covariate in all the main analyses. Of the 268 focal children with available BI data: 209 children at 24 months, 212 children at 36 months, 202 children at 48 months, and 197 children at 60 months also had available observational affect data. Missing data analyses on the BI and observational affect measures suggested that patterns of missing data did not violate the assumption that they were missing at random (MCAR), Little's MCAR, χ^2^ (9) = 10.07, *p* = .345.

### Aim 1: how much of children's affect expressions are accounted for by individual traits versus relational processes?

Actor, partner, and relationship variances all contributed to children's displays of positive and negative affect during their play with peers. Individual differences in children's tendencies to express positive and negative affect with peers accounted for 2.39% and 1.30% of the variance, respectively (i.e., actor variance). Play partners’ tendency to express positive and negative affect in a consistent way with a given focal child accounted for 0.01% and 0.23% of the variance, respectively (i.e., partner variance). Over and above these contributions, the unique relationship between the focal child and play partner accounted for 44.28% of the variance in positive affect and 47.50% of the variance in negative affect (i.e., actor relationship variance). Additionally, the way a specific play partner adjusted their affect expressions toward a given focal child explained a minor portion of the variance in children's positive (44.28%) and negative affect (50.22%) (i.e., partner relationship effect). Together, these indicate that children's expressions of positive and negative affect with peers are mostly due to the distinct nature of the relationship rather than the individual traits of each child.

### Aim 2: Are children's affect expressions correlated with their partners?

Focal children's and play partners’ expressions of positive affect (*r* = .45, *p* = .010) and negative affect (*r* = .34, *p* = .010) were moderately correlated. This indicates that children's affective expressions were concurrently associated with their partners’, such that, overall, higher levels of positive or negative affect in children were linked with higher levels of the same affect in their partners during free play.

### Aim 3: Does a child's behavioral inhibition relate to their own and their partners affect expressions?

#### Positive affect

Model 1 (see Table 2) examined whether focal children's BI was associated with their own and their play partner's positive affect. This model also included dyad gender, dyad age, both children's ethnicity, and the interaction between BI and dyad age. BI was inversely related to positive affect expressions during play, suggesting that focal children who were higher in BI than the sample mean displayed less positive affect with their partners. In contrast, focal children's BI was not related to their partners’ displays of positive affect. On average, boy dyads displayed more positive affect than girl dyads. Importantly, BI did not interact with dyad age, indicating that the association between BI and children's positive affect did not significantly vary by age. Older dyad age was related to higher levels of positive affect. Children own and their partner's ethnicity was not associated with their expressions of positive affect.

**Table 2:** Results of multi-level one-with-many models examining associations between actor behavioral inhibition and actor and partner observed positive affect and negative affect.

Fixed effect	Model 1: positive affect				Model 2: negative affect^a^			
	*B*	*SE*	95% CI	*R* ^2^	*B*	*SE*	95% CI	*R* ^2^
Focal Child Intercept	2.60**	0.11	[2.38, 2.82]		2.09***	0.11	[1.88, 2.30]	
Partner Intercept	2.56***	0.11	[2.34, 2.78]		2.09***	0.11	[1.88, 2.29]	
Dyad Age	0.03***	0.003	[0.02, 0.04]	.18	0.006*	0.003	[0.001, 0.01]	.01
Dyad Gender	0.33***	0.08	[0.17, 0.50]	.07	0.17*	0.08	[0.02, 0.32]	.02
Ethnicity on Own Affect	0.09	0.07	[−0.06, 0.23]	.00	−0.03	0.07	[−0.17, 0.12]	.00
Ethnicity on Partner's Affect	−0.03	0.07	[−0.17. 0.11]	.00	−0.14	0.07	[−0.28, 0.00]	.01
Focal Child BI on Own Affect	−0.30***	0.09	[−0.48, −0.12]	.01	−0.08	0.09	[−0.26, 0.10]	.00
Focal Child BI on Partner Affect	−0.09	0.07	[−0.27, 0.09]	.00	−0.15*	0.06	[−0.27, −0.03]	.01
Focal Child BI*Dyad Age	0.00	0.004	[−0.01, 0.01]	.00	0.001	0.004	[−0.01, 0.009]	.00

*Note. B*s represent unstandardized regression coefficients. Gender (0 = female 1 = male), children's ethnicity (0 = other ethnicities, 1 = White). aNegative affect was winsorized to remove outliers (≥ 3 from the mean). Results rerun without winsorizing showed no change in statistical significance or interpretation.

#### Negative affect

Model 2 (see Table 2) tested whether focal children's BI was related to their own and their play partner's negative affect. This model also included dyad gender, dyad age, both children's ethnicity, and the interaction between BI and dyad age. Focal children's BI was not associated with their own displays of negative affect during play across ages. In contrast, focal children's BI was inversely related to their play partners’ displays of negative affect, suggesting that focal children who were higher in BI than the sample mean had partners who displayed less negative affect. BI did not interact with dyad age, indicating that the association between BI and children's negative affect did not significantly vary by age. Older dyad age was linked with higher levels of negative affect. On average, boy dyads displayed more negative affect than girl dyads. Children own and their partner's ethnicity was not associated with their expressions of positive affect.

## Discussion

The way children express their emotions when they first meet a new peer is central to relationship formation. We employed a longitudinal dyadic design to examine the contributions of individual traits and the specific relationship on children's affect expressions when interacting with new peers across early childhood. We also investigated how behavioral inhibition relates to children's own and their partner's affect expressions. Three key findings emerged: (1) children's positive and negative affect were accounted for by both individual differences and relational processes, (2) children's affective expressions were concurrently associated with their partners’, and (3) children higher in BI expressed less positive emotion across play partners over time and elicited fewer displays of negative affect in their play partners in early childhood. Together, these findings indicate that temperament and relational context jointly shape children's emotional expressions during early peer interactions, with implications for how peer relationships begin to form in early childhood.

### Individual and dyadic contributions to children's emotional expressions with new peers

Our findings indicate that young children's emotional expressions with new peers were accounted for by both their dispositional tendencies and the unique qualities of each dyadic relationship. The relative contributions of the relationship to children's positive and negative affect expressions during play was greater than that of their individual traits. Importantly, these contributions were evident regardless of their age, gender, and their level of behavioral inhibition. The relationship-level contributions suggest that differences in children's affective exchanges reflect not only each child's characteristics but also the pairing of partners, pointing to the value of considering dyadic context in understanding peer interactions. Examples of dyadic context may include features that emerge from the pairing of two children, such as similarities or differences in temperament (e.g., two highly exuberant children amplifying each other's positive affect), play styles (e.g., one child leading while the other follows), or approach–withdrawal tendencies (e.g., an inhibited child paired with a socially eager peer). These findings align with interpersonal theories of development (Hinde, 1997) and recent empirical research. Specifically, Hubbard et al. (2023) found that school-aged children's emotional expressions were influenced by both the trait-like and the relational aspects of these expressions during structured dyadic tasks with new peers. An important caveat when interpreting our findings is that some peers participated in more than one dyad, meaning that partner and relationships effects may partly reflect characteristics of these peers rather than being fully independent. It will thus be important for future studies to replicate our findings with unique partners for each dyad. Collectively, these results highlight the need for future research to examine which dyadic features are linked with positive peer experiences and the early formation of friendships.

We also found that children's affective expressions were correlated with those of the unfamiliar peers they played with across early childhood. This echoes studies on emotional mimicry and behavioral synchrony demonstrating that children mimic and become in sync with the emotional expressions of parents, peers, and adult strangers (Feldman 2014; Hess & Fischer, 2014; Hubbard et al., 2023). This study is the first to examine the overall association between peer dyads affect expressions between 24 and 60 months of age, a period that is instrumental to the development of children's social and emotional skills (Halberstat et al., 2001). The act of matching positive affect may serve as an important signal of children's shared openness to friendship and enjoyment of the interaction to their peer, building the foundation for friendship formation (Fredrickson, 2001; Hess & Fischer, 2014). Research has indeed found that adults who mimic the emotions of others report liking each other more after their first interaction (Fujiwara et al., 2020). Conversely, when a child reciprocates negative affect, it may indicate a sense of shared distress or empathy within the interaction making its implications for friendship initiation more nuanced. It would be beneficial for future research to employ fine-grained, time-series approaches (e.g., second-by-second coding) to capture the temporal sequencing and mutual influence of children's emotional exchanges. Such methods would allow researchers to determine not only whether children's affective states align but also how one child's expressions shape the other's responses over time.

### BI and dyadic associations with positive and negative affect

The broaden-and-build theory of positive emotions proposes that positive affect broadens children's behaviours and builds resources (e.g., relationships), influencing children's later expressions of positive affect and relationship quality (Fredrickson, 2001; Ramsey & Gentzler, 2015; Stifter et al., 2020). We found that children higher in BI at 24 and 36 months displayed less positive affect at all ages of assessment irrespective of the specific unfamiliar social partner. Based on the broaden-and-build theory of positive emotions, we speculate that by expressing less positive affect children high in BI communicate fewer signals to their new social partners about their interest and openness to engagement, which may ultimately reduce the likelihood they will form an ongoing relationship. Aligning with this speculation, Taylor et al. (2017) found that increases in positive emotions during a relationship building exercise among adults with social anxiety disorder were associated with greater feelings of connectedness and stronger desires to engage with a previously unfamiliar partner in future social activities. Our findings also parallel those of a meta-analysis which demonstrated that social withdrawal is associated with less positive (but not more negative) friendship quality (Dryburgh et al., 2022). Ultimately, the blunted positive affect expressions of highly inhibited children during novel peer interactions may provide them with fewer opportunities to build up the positive aspects of friendships.

Contrary to our prediction, BI was not associated with children's own negative affect expressions across interactions with unfamiliar peers. This prediction was based on several studies that find that higher BI is associated with higher parent, teacher, and self-reported trait negative affect and higher observed negative affect with *one* unfamiliar peer (e.g., Eggum et al., 2012). In contrast to previous work, we measured negative affect repeatedly across different unfamiliar peers and early development. It may be that children high in BI are more inclined to display negative affect in some interaction but not others (e.g., with certain partners). Nonetheless, the present findings pertaining to negative affect should be interpreted with caution because of the low frequency and intensity of negative affect observed in the current sample across timepoints (*M* = 2.17 on a 7-point scale). Given the low frequency of displays of negative affect, a plausible interpretation may be that children implemented display rules and masked their negative emotion expressions with new peers. Indeed, research shows that children as young as 3 years old can control their expressions of disappointment by displaying less negative affect in the presence of others (e.g., Cole, 1986). According to Ekman et al. (1969), one strategy children may implement to mask their emotions is expressing neutral affect (e.g., a “poker face”). Research demonstrates that highly inhibited children lack spontaneity in their expressions (e.g., Kochanska & Radke-Yarrow, 1992) and that older children high in shyness show high levels of neutral affect when interacting with a new peer (Sosa-Hernandez et al., 2023). Based on this work, we further speculate neutral affective expressions may be one way highly inhibited children behaviorally express their vigilance toward new peers. Our study was limited by our use of global codes of positive and negative affect and did not code for neutral affect, and thus future work should test this hypothesis.

Interpersonal models of development emphasize the transactional effects of temperamental traits such as BI on children's own and their partner's behaviors (Hinde, 1997). Supporting this account, we found an that children higher in BI elicited *less* negative affect from their unfamiliar play partners (albeit with a small effect size). This coincides with the findings from Walker et al. (2015), which found that children higher in social fear at 24 months elicited less social engagement from their unfamiliar play partners at 36 months. Our finding extends this work by providing evidence of its continuity across early childhood and different unfamiliar social partners. BI, however, was not related to partner's displays of positive affect. One potential explanation for why BI was related to less peer negative affect but not to peer positive affect is that highly inhibited children often engage in avoidance or solitary play during novel interactions (Coplan et al., 2004; Walker et al., 2015). In these contexts, they may watch their partner or play separately, effectively allowing the peer to “do their own thing.” This may reduce opportunities for conflict, such as object struggles during social play to emerge, while still leaving the peer free to enjoy their own play activities. Future research should examine whether these patterns replicate in other samples and test potential mechanisms, such as children's engagement in shared versus independent play, that may help explain why young children high in BI elicit less negative but not less positive affect from peers during their first play encounters. Collectively, our findings demonstrate that BI influences both children's displays of emotions in nuanced ways, limiting the quality of young children's initial peer interactions.

Characteristics of the dyad were also associated with children's affective expressions during new peer interactions. For example, we found that dyads composed of two boys showed higher levels of both negative and positive affect, and that the frequency and intensity of affective expressions increased with age. These findings likely reflect developmentally appropriate changes in the nature of play, such as transition from parallel play to sociodramatic play, that increase the likelihood that children will in general engage in affectively charged interactions, for better or for worse. Our findings suggested that children's ethnicity was not associated with their affective behavior during novel peer interactions. However, our results should be interpreted cautiously given the limited ethnic diversity of our sample (i.e., 70% White) and the coarse measurement of ethnicity (White vs. Other Ethnic groups). Especially, given prior research indicating that North American cultural contexts often emphasize overt displays of positive affect, whereas other contexts may value emotional restraint or prioritize relational harmony over expressiveness (Halberstadt et al., 2001; Matsumoto, 1993). Future research with more diverse samples should investigate whether cultural norms and emotion socialization practices may moderate these affective processes. It will also be interesting to consider the interplay between actor and partner ethnicity, as children may adjust their affective displays based on shared versus different cultural backgrounds or in response to perceived bias or cultural competence.

### Limitations and future directions

There are several limitations that should be noted when interpreting the results of the current study. Our sample was relatively homogenous in terms of ethnicity and education which may tamper the generalizability of our findings. It will be important for future work to replicate the present findings using more ethnically and economically diverse samples, especially given cultural variations in how shyness is displayed and interpreted, as well as how emotions are socialized and expressed (Chen, 2019; Matsumoto, 1993). Another limitation concerns our handling of missing data. More robust methods such as full information maximum likelihood estimation or multiple imputation could not be applied because they do not accommodate the complex random-effect variance components that are central to our analyses. Nevertheless, the non-significant Little's MCAR test suggests that our missingness was completely random, which means our parameter estimates are likely unbiased, though our statistical power to detect significant effects may be reduced. We also used children's displays of emotions as behavioral indicators of the quality of the interaction; however, our understanding of impression formation processes would have been enhanced by the inclusion of children's reports of their liking toward the new partner. It will also be critical for future research to use repeated interactions with the same peer to further understand how affect dynamics predict the transition from acquaintanceship to friendship. Another exciting direction for future research would be to assess the BI of both dyad members to examine how similarity or differences in BI shapes affective exchanges during children's first peer encounters. Finally, given the multi-faceted nature of emotions, it will be crucial for future work to replicate our using more discrete emotion categories (e.g., anger, nervousness) and other indicators of emotion (e.g., physiological arousal).

## Conclusion

By approaching emotions through relational framework, our study showed that young children's emotion expressions when meeting a new peer are driven by both their individual traits and the specific relationship. In particular, the unique relationship between peers made substantial contributions to children's expressions of affect, highlighting how important it is to understand the dyad specific factors that can make it easier or harder for children to become friends. We also found that children's emotions were generally related to those of their play partners, suggesting an overall shared emotional tone during their interactions. Additionally, our study shows that children's individual characteristics including age, gender, and behavioral inhibition relate to how they express affect with new peers. We found that children high in behavioral inhibition show less positive affect themselves and elicit less negative emotions from new play partners across early childhood. These findings suggest that the success of early social interactions between children may stem from the combination of their individual traits and attributes of the relationship.

## Data Availability

The analytic code necessary to reproduce the analyses presented here is available from the first author upon request. The data and materials necessary to reproduce or replicate the findings are not publicly accessible due to participant confidentiality restrictions. The analyses presented here were not preregistered.
